# Immunogenicity of the outer domain of a HIV-1 clade C gp120

**DOI:** 10.1186/1742-4690-4-33

**Published:** 2007-05-17

**Authors:** Hongying Chen, Xiaodong Xu, Ian M Jones

**Affiliations:** 1 School of Biological Sciences, The University of Reading, Reading, RG6 6AJ, UK

## Abstract

**Background:**

The possibility that a sub domain of a C clade HIV-1 gp120 could act as an effective immunogen was investigated. To do this, the outer domain (OD) of gp120_CN54 _was expressed and characterized in a construct marked by a re-introduced conformational epitope for MAb 2G12. The expressed sequence showed efficient epitope retention on the isolated OD_CN54 _suggesting authentic folding. To facilitate purification and subsequent immunogenicity OD_CN54 _was fused to the Fc domain of human IgG1. Mice were immunised with the resulting fusion proteins and also with gp120_CN54_-Fc and gp120 alone.

**Results:**

Fusion to Fc was found to stimulate antibody titre and Fc tagged OD_CN54 _was substantially more immunogenic than non-tagged gp120. Immunogenicity appeared the result of Fc facilitated antigen processing as immunisation with an Fc domain mutant that reduced binding to the FcR lead to a reduction in antibody titre when compared to the parental sequence. The breadth of the antibody response was assessed by serum reaction with five overlapping fragments of gp120_CN54 _expressed as GST fusion proteins in bacteria. A predominant anti-inner domain and anti-V3C3 response was observed following immunisation with gp120_CN54_-Fc and an anti-V3C3 response to the OD_CN54_-Fc fusion.

**Conclusion:**

The outer domain of gp120_CN54 _is correctly folded following expression as a C terminal fusion protein. Immunogenicity is substantial when targeted to antigen presenting cells but shows V3 dominance in the polyvalent response. The gp120 outer domain has potential as a candidate vaccine component.

## Introduction

The need for a form of HIV-1 envelope protein capable of eliciting a broadly neutralising antibody (NAb) response as part of an HIV vaccine has been widely discussed [1-3]. It is generally agreed that the lack of NAb is a consequence of a number of evasion mechanisms evolved by the virus to maintain immunological silence. Examples include glycan shrouding [4] and envelope structural heterogeneity [5,6]. Envelope structural heterogeneity involves primarily the outer envelope protein gp120. The monomeric molecule is flexible, with comparisons between the crystal structures of liganded and unliganded gp120 showing significant local change [7,8]. Of the three defined structural domains, the inner domain, bridging sheet and the outer domain (OD), both the inner domain and bridging sheet rearrange substantially upon CD4 binding [8]. The inner domain and bridging sheet are also the source of heterogeneity within monomeric gp120 in solution [6] and have sufficient flexibility to allow structural complementation between adjacent molecules [9,10]. There is additional heterogeneity in the envelope molecules on the virion surface where gp120 and transmembrane gp41 make up the virion spike which appears patchily distributed and in a variety of conformations [5,11,12]. Strategies for improving gp120 immunogenicity have been reviewed recently [13]. One approach has examined the gp120 OD in isolation as, by contrast with the complete molecule, it is structurally stable [14]. The OD is heavily glycosylated and relatively immunologically silent in infected individuals but its potential to act as a generator of NAb is demonstrated by the fact that the epitope for a broad ranging, neutralizing human monoclonal antibody, 2G12, maps to it [15,16] as do a number of lectins which potently neutralize virus infectivity *in vitro *[17,18]. MAb 2G12 is unique in that recognition of its epitope, a high-mannose carbohydrate cluster on gp120, is achieved through a dimeric antibody structure in which there is V_H _exchange between adjacent immunoglobulin molecules [19]. The paucity of this form of Ig structure in total IgG [20] suggests that a similar specificity may be difficult to generate following immunisation but, as the juxtaposition of key N-linked glycans is essential for MAb binding, the 2G12 epitope does provide a sensitive measure of OD conformation. Yang *et al.*, showed that the OD (residues _252_RPVVST.....DNWRS_482_) of gp120 from B clade virus YU2, which they termed OD1, retained 2G12 binding despite being poorly detected by the majority of HIV positive sera [14,21]. Interest in the domain lies in the fact that 2G12 does not directly compete for the primary receptor binding site on gp120 [19] but appears to impair secondary receptor binding [22]. In addition, gp120-2G12 complexes exhibited reduced binding to DC-SIGN, consistent with antibody capping of at least some of the mannose moieties that would otherwise bind the lectin [22]. Such *in vitro *inhibition of gp120 receptor binding is of consequence *in vivo *as 2G12, in combination with other neutralizing MAbs, such as b12 (against the CD4 binding site [23]) and 2F5 (against gp41), provides protection against HIV-1 challenge in animal models [24-26]. M-type HIV-1 includes 9 clades [27-29] and vaccine candidates designed for beneficial antibody induction should provide immunity against all clades if they are to be effective [3]. It has been noted that C clade envelopes are significantly different to those of B clade isolates, especially early in infection [30,31] and, more generally, that subtype C candidate vaccine development has been reportedly more problematic than clade B focused strategies [32]. HIV-1 C clade isolates have frequently lost the carbohydrate sites required by 2G12 and so rarely present the epitope [33]. It was unclear therefore if data obtained with the OD of the B clade gp120_YU2 _[14] would be repeated using the OD of a C-clade isolate nor yet how a meaningful conformation of the latter OD could be confirmed in order to test such a possibility. Here, using the gp120 sequence of HIV-1_CN54_, a B/C clade recombinant originally isolated in China [34,35], we examine the C clade gp120 OD using a re-engineered 2G12 epitope to provide a measure of conformational relevance before use as an immunogen. Subsequently, Fc tagged OD is used as an immunogen in a comparative study with gp120-Fc and untagged gp120.

## Results and discussion

### Characterisation of the 2G12 epitope reconstructed in clade C gp120

**Figure 1:**
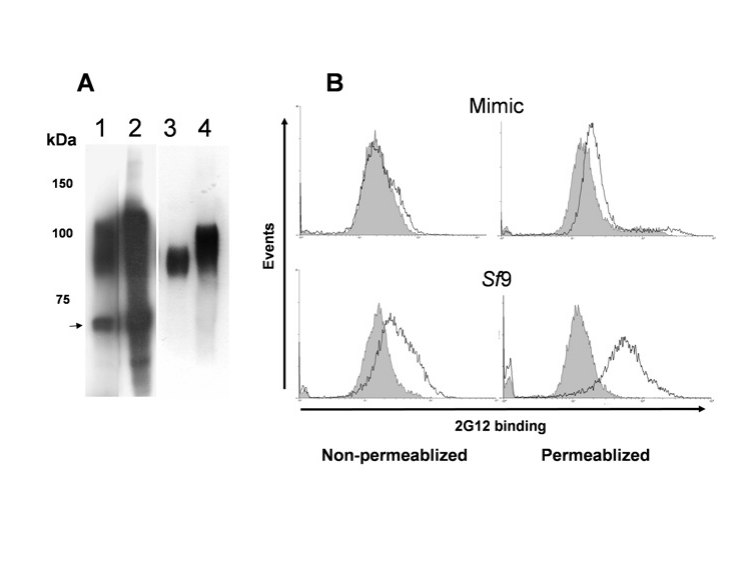
Characterisation of HIV-1 gp120_CN54 _(V295N+A394T) expressed as a transmembrane bound protein in both *Spodoptera frugiperda *(Sf9) and Mimic cells (Invitrogen). **A**. Western blot of cell extracts (tracks 1 and 2) and supernatant (tracks 3 and 4) showing the increase in size associated with gp120 with modified glycans. Track 1 and 3 – *Sf*9 cells, Track 2 and 4 – Mimic cells. Note the presence of the unglycosylated apoprotein (arrowed) and glycosylation intermediates in the cell extracts which are absent in the supernatant. The gp120 present in the supernatant is due to pickup by budding baculovirus particles (see [53]). Markers to the left are in kilodaltons. **B**. Flow cytometry of permeablized or non-permeablized *Sf9 *and Mimic cells following staining with 2G12. Cells were permeablized by incubation in 0.1% Triton X-100 followed by fixing in 3% paraformaldehyde. Control cell profiles are shaded, infected cell profiles are unshaded.

**Figure 2:**
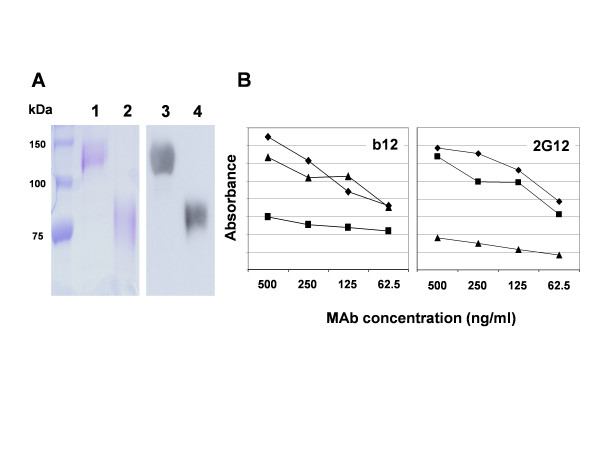
Purification of gp120_CN54_^+^-Fc and OD_CN54_^+^-Fc and reaction with MAbs b12 and 2G12. **A**. Protein present in the supernatant of recombinant baculovirus infected insect cells was purified by lectin and protein A chromatography and analysed by SDS-PAGE (tracks 1 and 2) and western blot (tracks 3 and 4) using an anti-human Ig conjugate. Tracks 1 and 3 – gp120_CN54_^+^-Fc;Tracks 2 and 4 – OD_CN54_^+^-Fc. Protein size markers indicated to the left are in kilodaltons (kDa). **B**. Purified protein was coated onto plastic at 10 μg/ml and used to assess the binding of monoclonal antibodies b12 or 2G12 as shown. The samples were (▲) – gp120_CN54_^+^-Fc; (■) – OD_CN54_^+^-Fc; (◆) – gp120_CN54_^+^. Binding was detected by an anti-human Ig light chain conjugate.

Despite the sequence variation between HIV B and C clade gp120 [29] reintroduction of two glycosylation triplets centred on the asparagine residues 295 and 392 (actual mutations V295N+A394T) was sufficient for MAb 2G12 recognition to the C clade HIV-1 envelope protein gp120_CN54_. Reintroduction of either site alone did not allow significant recognition [36]. Before assessing if the 2G12 epitope could be presented on the OD of gp120_CN54 _we assessed the nature of the 2G12 site re-engineered into gp120_CN54 _in more detail. The 2G12 epitope is mannose [15,16,21] presented in such a way that it provides specificity for gp120. When expressed in insect cells gp120 is naturally highly mannosylated [37] but the use of insect cells engineered to express glycotransferases capable of adding further sugar moieties characteristic of mammalian cells [38,39] leads to mature glycans terminating in galactose and N-acetylglucosamine. Intracellular forms of the glycoprotein, yet to complete trimming and modification, remain terminally mannosylated. To assess the role of terminal mannose in the recognition of the 2G12 epitope re-engineered into gp120_CN54 _(hereafter referred to as gp120_CN54_+), a transmembrane form of gp120_CN54_+ [36] (additional file 1A) was expressed in both *Spodoptera frugiperda *(Sf9) cells and Mimic cells (Figure 1A) and 2G12 reactivity assessed by flow cytometry before and after cell permeablisation. Sf9 cells expressing gp120_CN54_+, showed excellent reactivity with MAb 2G12 in both non-permeablised and permeablised formats (Figure 1B) indicating folded mannosylated protein on the cell surface. However, while gp120_CN54_+ expressed in Mimic cells reacted with 2G12 in the permeablised format it failed to react when cells were not permeablised (Figure 1B) despite the detection of gp120 on the cell surface by staining with polyvalent serum ADP423 [40]. This data confirms the expected phenotype of the re-introduced 2G12 epitope where terminal mannose on the glycans attached to asparagines 295 and 392 is required for MAb binding.

### The 2G12 epitope is maintained on the isolated outer domain

The juxtaposition of mannose residues on the termini of key gp120 glycans creates the 2G12 epitope which is therefore conformational [15,16,21,33]. Yang *et al.*, described maintenance of the 2G12 epitope on the OD of HIV-1_YU2 _[14] which also defined the OD as conformationally relevant. To assess if this was also true of the re-engineered 2G12 epitope on gp120_CN54_+, the OD of gp120_CN54 _(residues_251_IKPV...NWRS_481_) was amplified from a clone encoding gp120_CN54_+, cloned (additional file 1B)

and expressed as a human Ig Fc tagged protein using recombinant baculoviruses [41,42]. Fc tagged gp120_CN54_+ was also expressed and purified to provide a control. Fc tagging at either terminus of gp120 to provide a simple means of purification and detection has been also used elsewhere [22,43]. Proteins present in the supernatant of infected insect cells at two day post infection were purified by successive chromatography on lectin (*lens culnaris*)-sepharose and protein A-sepharose. Purified OD_CN54_+-Fc protein migrated as a single band of ~85 kDa by SDS-PAGE whereas the gp120_CN54_+-Fc migrated at ~130 kDa consistent with addition of the Fc domain (~25 kDa) to the gp120 sequence in each case (Figure 2A). Both proteins were dimers under non-reducing conditions (not shown). Unfused gp120_CN54_+ has been described previously [36]. Proteins were normalised by OD_280 _and reaction with the polyvalent antisera ADP423 prior to assay by ELISA to assess 2G12 and, for gp120_CN54_+ and gp120_CN54_+-Fc, b12 binding. The purified complete gp120 proteins bound to b12 with equivalent efficiency suggesting the C-terminal Fc tag had no effect on the primary receptor binding conformation whereas OD_CN54_+-Fc bound b12 poorly (Figure 2B). The b12 epitope has been recently shown to lie within OD, at least of B clade isolate [23]. However, in the absence of the inner domain, the dissociation rate for the b12-OD complex is increased 15 fold when compared to b12-gp120 [23]. This likely explains the poor b12 binding to OD_CN54 _in the ELISA format used here although a slightly different footprint for b12 on the C compared to B clade antigen cannot be ruled out. By contrast 2G12 binding was equivalent for gp120_CN54_+ and OD_CN54_+-Fc but was much reduced for gp120_CN54_+-Fc (Figure 2B). A reduction in 2G12 binding was not observed when Fc was fused to the N-terminus of gp120 although DC-SIGN binding was affected by the location of the Fc tag [22]. We conclude that Fc fusion to the C terminus of complete gp120 may partly occlude the 2G12 binding site but that the reconstructed 2G12 epitope is exposed on the isolated CN54 OD.

### Immunogenicity of OD_CN54_^+^-Fc, gp120_CN54_^+ ^and gp120_CN54_^+^-Fc

**Figure 3:**
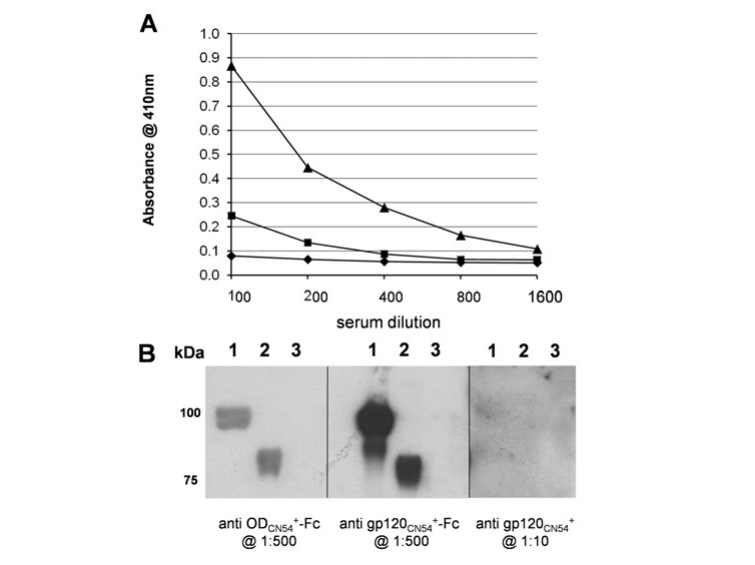
Analysis of serum response to various CN54 gp120s by ELISA and Western blot. **A**. Pooled mouse sera were assayed on highly purified CN54 gp120 in a reciprocal dilution series starting at 1:100 and binding detected with an anti-mouse conjugate. The immunogens were: (▲) – gp120_CN54_^+^-Fc;(■) – OD_CN54_^+^-Fc;(◆) – gp120_CN54_^+^. **B**. Western blot analysis of each serum on gp120_CN54_^+ ^(track 1) OD_CN54_^+^-Fc (track 2) and gp120_Bal _(track3 – to assess cross clade reactivity). The panels were blotted with the sera raised to the proteins shown and were used at the dilutions indicated. Anti-Fc reactivity in the sera was blocked by pre-incubation with excess HCV E2-Fc (not described).

**Figure 4:**
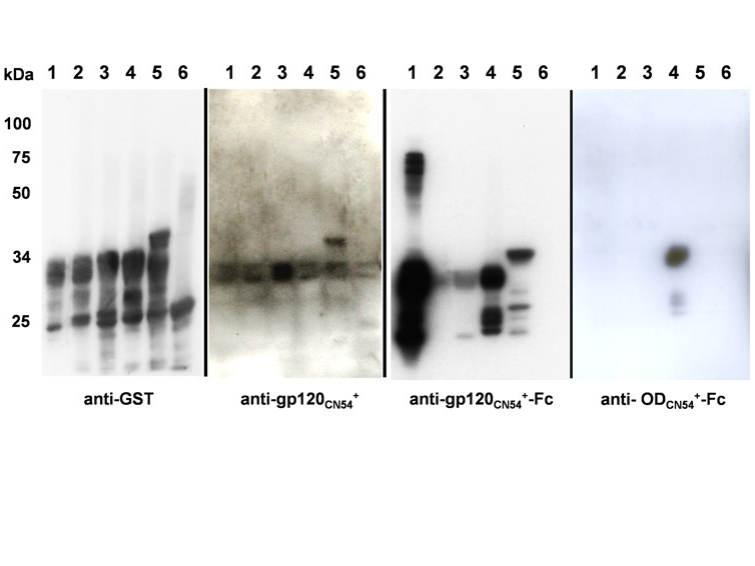
Analysis of serum response to GST-gp120_CN54 _fragments by Western blot. Expression of each GST fusion protein was induced for 3 hrs and the equivalent of 50 μl of the culture fractionated on 10% SDS-PAGE before the transfer. Lanes 1–5 are the GST-gp120_CN54 _fusion proteins 1 to 5 with the origin of the gp120_CN54 _fragments as shown in additional file 2. Lane 6 is GST only (western blot reaction at 26 kDa and 54 kDa in panel A are monomer and dimer of GST respectively). The GST serum (Sigma) was used at 1:5000, serum dilutions for blots B-D were as described for figure 3.

Purified protein, 10 μgs per injection, was used to immunise groups of mice (n = 3) in the absence of adjuvant as the Fc domain has adjuvant activity [44]. In all, three immunisations were made at two week intervals and the animals bled two weeks after the final immunisation. The sera were pooled and assayed by ELISA for titre against purified gp120_CN54_+ and for breadth of response by Western blot on five overlapping fragments of gp120_CN54 _expressed as GST fusion proteins in *E. coli*. The fragments used encoded residues 34–129 (the C1 domain), 117–207 (the V1V2 domain), 192–302 (the C2 domain), 289–394 (the V3C3 domain) and 358–510 (the V4-C5 domain) (additional file 2). As expected, gp120_CN54_+ in the absence of adjuvant was very poorly immunogenic even after repeated immunisation. However, both Fc fusions elicited significant gp120 titres with gp120_CN54_+-Fc providing a higher titre than OD_CN54_+-Fc (Figure 3). When used to blot the overlapping GST gp120_CN54 _fragments, serum raised to gp120_CN54_+ showed weak reactivity to fragment 5 (V4-C5) while the gp120_CN54_+-Fc serum showed the strongest reaction with fragment 1 (C1 domain) followed by fragments 4 and 5 (V3C3 and V4-C5 respectively) (Figure 4). There was only modest reactivity with fragments 2 and 3 representing the V1V2 and C2 domains. Immunisation with OD_CN54_+-Fc resulted in a predominant anti V3C3 response with minor reactivity on the V4-C5 fragment (Figure 4). Serum reactivity was also assessed by ELISA and found to be similar suggesting that little of the immune response was to conformational epitopes (additional file 3). However, the use of antigens expressed in bacteria may have limited the detection of conformational antibodies or those directed wholly or partly to epitopes that include carbohydrate.

**Figure 5:**
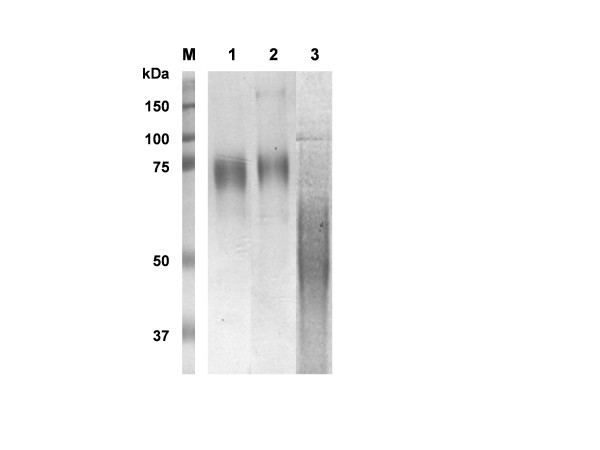
Purification of OD_CN54_+-Fc, OD_CN54_+-Fc(VA) and OD_CN54_+-His. Protein present in the supernatant of recombinant baculovirus infected insect cells was purified by a combination of lectin and protein A chromatography and analysed by 10% SDS-PAGE. Track M – markers with molecular weights as shown; track 1 – OD_CN54_+-Fc; track 2 – OD_CN54_+-Fc(VA) and track 3 – OD_CN54_+-His. Note the slightly slower migration of the VA mutant as a result of the hydrophobicity change in the loss of two leucines within the Fc coding region. The smear of OD_CN54_+-His was typical and reflects the high level of glycosylation on a fairly small protein fragment. The yield for all constructs was ~1 mg/L of culture.

**Figure 6:**
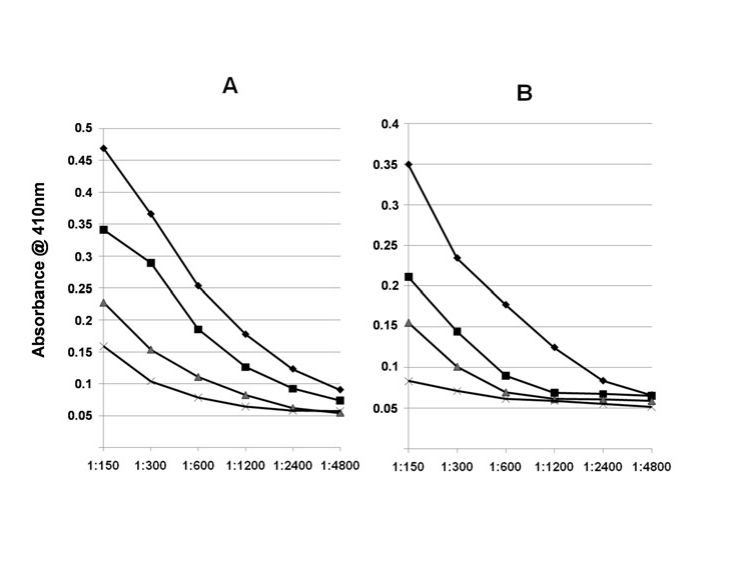
Analysis of serum responses to gp120_CN54 _(A) and OD_CN54 _(B) assessed by ELISA following immunization with the various OD_CN54_constructs described. Protein immunogens, as shown in figure 5 plus the complex between OD_CN54_+-Fc and GG7, were used to immunise groups of mice as before and terminal serum titres obtained following incubation on immobilised gp120_CN54 _and OD_CN54 _followed by an anti mouse conjugate. The sera were generated to: OD_CN54_+-Fc (◆); OD_CN54_+-Fc + GG7 (■); OD_CN54_+-Fc(VA) (▲) and OD_CN54_+-His (X).

The CN54 gp120 outer domain was rendered immunogenic by fusion to the Fc domain, which directs antigen uptake via murine IgG Fc receptors [45]. This type of complex has been shown previously to result in up to 10,000 fold enhancements of titre for proteins, including HIV proteins, expressed in plants [46,47]. However, whether this was entirely dependent on FcR mediated uptake or in part due to oligomerisation of the antigen via the Fc region was unclear. We sought to distinguish between these possibilities by further immunisations using solely the gp120 outer domain fused or not to Fc. The basis for FcR binding by Fc resides in selected residues in the CH2 domain of IgG1 [48,49]. We mutated two of the most significant residues, L_234_L_235_, to valine and alanine respectively (additional file 1C) and re-expressed and purified OD_CN54_+-Fc(VA) (Figure 5). In addition we expressed OD_CN54_+ without a Fc tag but tagged at the C terminus with poly-histidine (OD_CN54_+-His) to enable similar levels of purification (Figure 5). Lastly we formed larger oligomeric complexes of OD_CN54_+-Fc by cross linking the Fc domain through incubation with the anti human MAb, GG7 (Sigma Aldrich), in a 2:1 (OD_CN54_+-Fc:MAb) ratio. Complex formation was verified by analytical gel filtration chromatography although no attempt was made to fractionate a particular size class (data not shown). All OD preparations were quantified and used to immunise groups of mice as before, without adjuvant, and the pooled serum was assessed for reactivity with each antigen by both ELISA and Western blot. As for untagged gp120_CN54_+, OD_CN54_+-His was a very poor immunogen and raised sera that reacted weakly by ELISA with both gp120_CN54_+ and OD_CN54_+-His (Figure 6). As before however, fusion to the Fc tag resulted in the generation of substantial titres against both antigens (Figure 6). Complex formation, by the addition of anti human MAb GG7, did not further enhance immunogenicity and immunisation with OD_CN54_+-Fc(VA) led to reduced titres on both antigens (Figure 6). Similar data were apparent by western blot (additional file 4) and blots on the overlapping fragments of gp120_CN54 _expressed as GST fusion proteins showed the same predominant profile of interaction with the V3C3 fragments (cf. Figure 4) (data not shown).

This data confirms that the outer domain of gp120 in isolation can be immunogenic despite not normally generating significant responses in HIV-1 infected individuals. Similarly, the finding that the majority of the response was to polypeptide not carbohydrate despite the hyperglycosylation of the OD [14] is supported by the reaction of the sera generated with GST-gp120 fusion proteins expressed in bacteria. However, our data do not confirm the conclusion that the OD of HIV-1_YU2_, upon immunisation, resulted in few antibodies to the V3 loop [14]. That conclusion was inferred from serum depletion experiments and no direct epitope mapping was done whereas our use of overlapping fragments of gp120_CN54 _as antigen suggests that, for the OD_CN54_+, induction of a strong response to the V3C3 region of gp120 took place. The pattern of reactivity was the same irrespective of the titre of the serum obtained suggesting that this region is a prominent feature of the CN54 sequence.

The finding that OD-Fc fusions are improved immunogens is consistent with a mechanism of enhanced immunogenicity through engagement of the FcR on antigen presenting cells. The reduction in titre following immunisation with OD_CN54_+-Fc(VA) supports this conclusion as does the lack of enhancement by further oligomerization through GG7 cross linking. Although reduced, the serum titre in response to OD_CN54_+-Fc (VA) was still far higher than OD_CN54_+-His alone probably as a result of a residual level of FcR binding. Within the context of IgG1 the VA mutation leads to a 10–100 fold reduction in FcR binding [48] effectively meaning that 1–10% of the OD_CN54_+-Fc(VA) immunogen was capable of generating a serum response which was observed to be ~50% of the level obtained with the standard dose of non mutated OD_CN54_+-Fc. This suggests that gp120-Fc fusions would be immunogenic at significantly lower doses than those used here (10 μg), an important consideration in vaccine design.

The response to gp120_CN54_+-Fc included reaction with all 5 fragments probed but a predominant response against the N terminus (the C1 domain) and a fragment encoding the V3 loop. Reaction with the C1 domain is consistent with the flexible gp120 inner domain which stimulates antibodies that are non-neutralising [23]. The OD of the C clade gp120_CN54_+ presented a 2G12 epitope that bound MAb as well as the full length protein (or equivalent amounts of a B clade gp120 – not shown). As the spatial juxtaposition of glycans on residues 295 and 392 is crucial for MAb binding we infer from this that the OD of gp120_CN54_+ may be structurally very similar to that of the solved B clade structures [7,8,23]. Novel mannosylated compounds able to bind 2G12 have been suggested as synthetic immunogens that may be able to elicit 2G12 like antibody responses [50,51]. A framework based on the natural target, the gp120 OD domain, would be preferable if immunogenicity could be enhanced and the Fc fusion reported here is a simple method of achieving this with the aim of generating further antibodies with 2G12 like properties. In addition, reaction with the V3C3 region was substantial suggesting that substitution of the V3 loop within OD_CN54 _with other conserved neutralising epitopes, such as those from gp41, may allow their prominent exposure for the generation of broadly cross clade neutralising Abs as has been attempted recently by grafting into the V1/V2 region of gp120 [52].

## Conclusion

The 2G12 epitope is faithfully reconstructed in a clade C gp120 backbone with 2 glycosylation site additions. The epitope is retained on the C clade outer domain where it acts as a marker of outer domain conformation. A C-terminal Fc tag provided for enhanced immunogenicity in the absence of any other adjuvants for full length C clade gp120 and for the normally immunologically silent outer domain. The serum response to the outer domain indicated a prominent V3C3 presentation for the clade C molecule in contrast to what has been observed for the clade B outer domain. Conformationally defined gp120 fragments as immune complexes may have potential as part of a vaccine design strategy targeting humoral immunity.

## Methods

### Cells and manipulations

*E. coli *Top10 was used for the propagation of plasmids and all cloning. Expression using recombinant baculoviruses used *Spodoptera frugiperda *(*Sf*9) insect cells unless otherwise stated. *Sf*9 cells were cultured in SF900-II (Life Sciences) at 28°C. A description of the cloning and mutagenesis steps is shown in additional file 1.

### Recombinant baculovirus infections

Infections for virus growth were done at an MOI of 0.1 and for protein expression, an MOI of 3. Virus growth was typically for 4 days or until there was considerable cytopathic effect. *Sf*9 cells infected for protein expression were harvested 72 hours post infection and the glycosylated protein present in the supernatant purified as described.

### ELISA

Microtitre plates (Thermo Labsystems) were coated with purified proteins previously normalised, blocked with PBS, 5% dried milk powder and used immediately. Primary antibodies were incubated with antigen for 60 min at room temperature. Unbound antibody was removed by washing five times with PBS containing 0.05% v/v Tween-20 and the plate was incubated with HRP-conjugated anti-mouse antibody (1:1000, Chemicon) for one hour at room temperature. The plate was washed extensively and incubated with 3,3',5,5'-Tetramethylbenzidine (TMB) chromagenic substrate (Europa Bioproducts). The reaction was stopped by addition of an equal volume of 0.5 M HCl and the absorbance was read at 410 nm.

### Western blotting

Protein samples were separated on pre-cast 10% Tris.HCl SDS-polyacrylamide gels (BioRad) and transferred to Immobilion-P membranes (Millipore) using a semi-dry blotter. Filters were blocked for one hour at room temperature using TBS containing 0.1% v/v Tween-20 (TBS-T), 5% w/v milk powder. Primary antibody was used at a dilution of 1:500 in PBS-T, 5% w/v milk powder unless otherwise stated. Following several washes with TBS-T the membranes were incubated for 1 hour with HRP-conjugated anti-mouse antibody (Chemicon) and the bound antibodies detected by BM chemiluminescence (Roche).

## Competing interests

The author(s) declare that they have no competing interests.

## Authors' contributions

HC and XX developed and characterised the tagged expression sources and HC carried out the immunological analysis. IMJ conceived, planned and advised throughout the experimental study. All authors discussed the manuscript which was written by IMJ.

## Supplementary Material

Additional File 1 The genetic constructions used to study the reintroduced glycosylation sites in the background of CN54 gp120. **A**. The gp120 coding sequence was first cloned into pAcVSVG™ [53]. The mature gp120 coding sequence was truncated part way through the convertase cleavage site (REKR) to prevent removal of the VSV TM domain by late golgi cleavage. The site and identity of the mutations introduced at 295 and 394 are shown. **B**. gp120_CN54_+ and the outer domain derived from it were cloned into the vector pAcFc to enable expression as secreted, Fc tagged molecules. The junctions of the fragments used are shown.**C**. Site directed mutagenesis of the Fc domain to reduce binding to the Fc receptor through change of the double leucine motif. The location and residues changed are shown. gp64 sig – the signal sequence from the major baculovirus surface glycoprotein gp64. VSVG™ – the transmembrane domain of Vesicular Stomatisis Virus. Ph – polyhedrin. Hu Ig Fc – human IgG1 Fc domain. Click here for file

Additional File 2 Glutathione-S-transferase fusion proteins expressed in *E. coli *to provide broad epitope mapping. **A**. Cartoon of the general construction, the vector used was pGEX2T. **B**. Schematic of the fragments amplified, cloned and expressed based on the widely used secondary structure model of gp120 originally described by Leonard *et al., *[54]. Click here for file

Additional File 3 Binding of the sera generated in this study to GST-gp120 fusion proteins by ELISA. Induced cultures expressing each of the GTS-gp120 fusion proteins were lysed by a mix of lysozyme and Triton and the fusion protein present captured and purified using Microspin GST columns (Amersham Biotech). Eluted fusion proteins at 10 μg/ml in 0.2 M NaHCO_3 _were used to coat the plate. Each serum was titrated on each construct and the endpoint titre determined. The relative binding of each serum to each fragment at this titre of serum is shown. Peak height s does not therefore represent their overall relative titre. Click here for file

Additional File 4 Western blot of various sources of CN54 gp120 by the mouse sera raised by immunisation with only the outer domain, fused or not to Fc. The sera were used at the single dilution shown where blot intensity broadly paralleled the titre obtained by ELISA (Figure 6). Reaction between the serum raised by immunisation with OD_CN54_+-His and the targets was very poor and required a tenfold less dilution than the others. Reaction with OD_CN54_+Fc appears stronger as the band is much tighter (cf. Figure 5) although reaction with the smear of the cognate antigen is just visible (rightmost panel track 3). Click here for file
