# Supplementary figures and images for: Immunogenicity of the outer domain of a HIV-1 clade C gp120
Source: Retrovirology. 2007 May 17;4:33. doi: 10.1186/1742-4690-4-33 (PMC1891314; doi:10.1186/1742-4690-4-33)

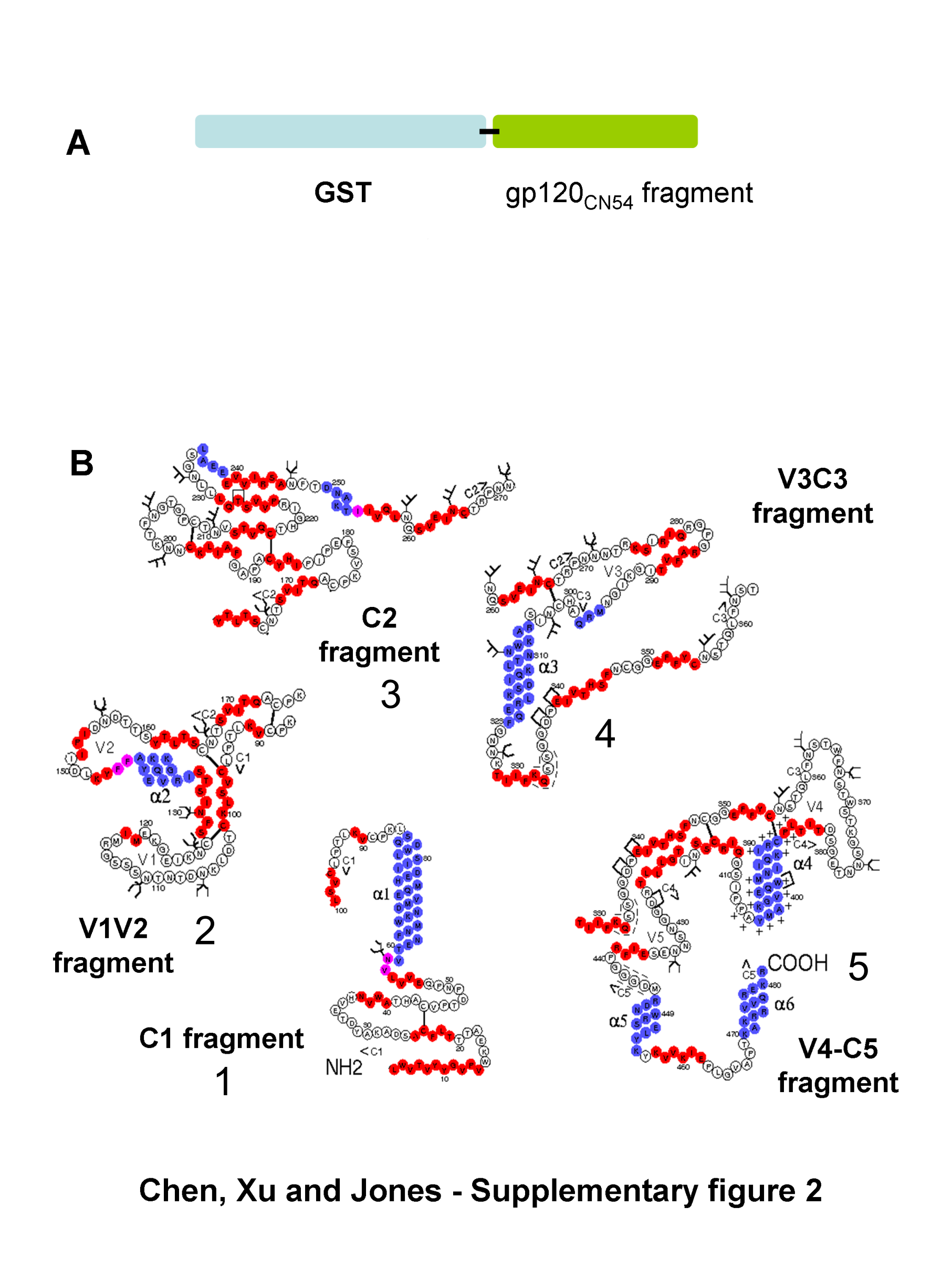

Supplement: Additional File 2 — Glutathione-S-transferase fusion proteins expressed in E. coli to provide broad epitope mapping. A. Cartoon of the general construction, the vector used was pGEX2T. B. Schematic of the fragments amplified, cloned and expressed based on the widely used secondary structure model of gp120 originally described by Leonard et al., [54]. [file 1742-4690-4-33-S2.tiff]

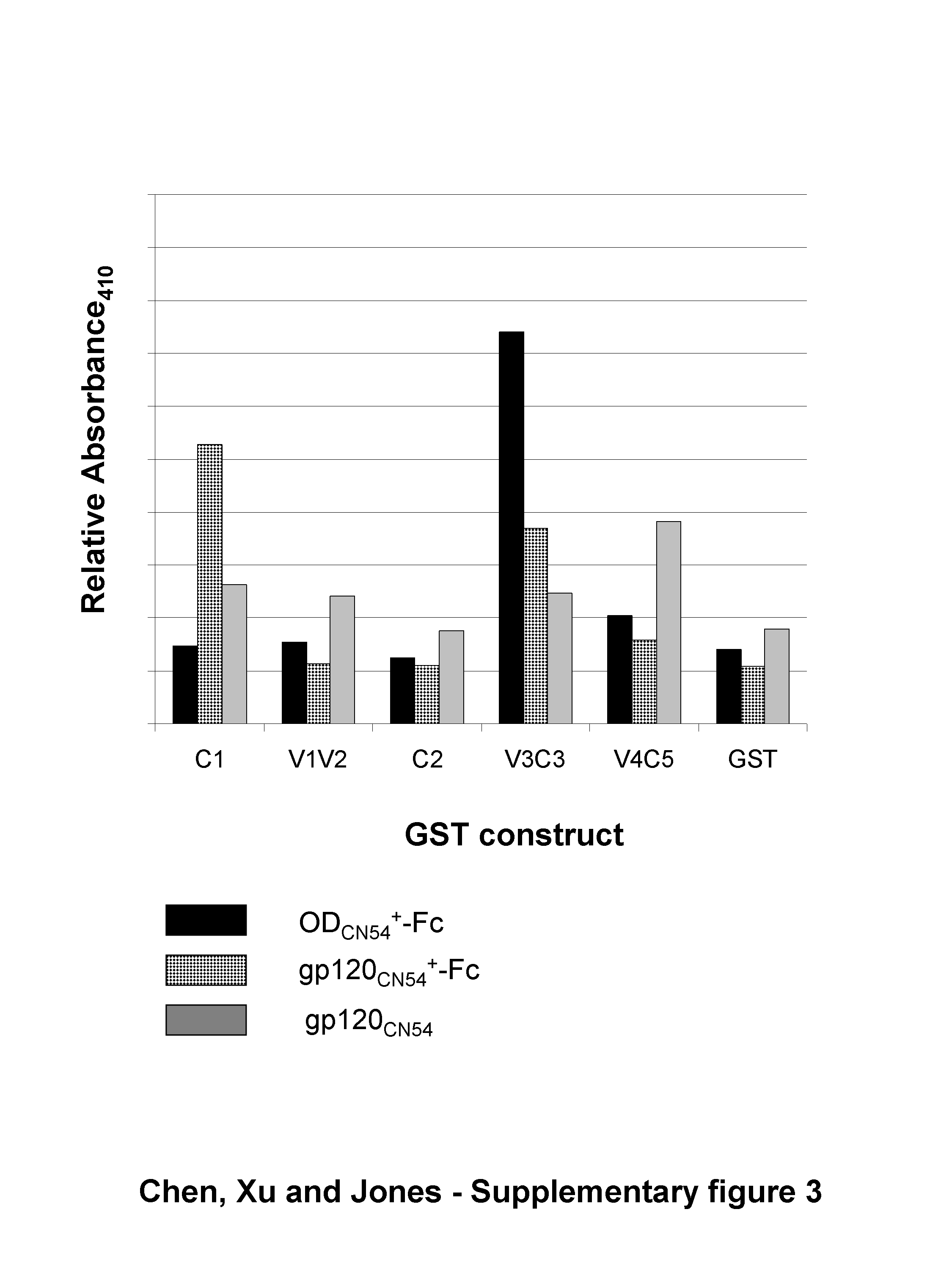

Supplement: Additional File 3 — Binding of the sera generated in this study to GST-gp120 fusion proteins by ELISA. Induced cultures expressing each of the GTS-gp120 fusion proteins were lysed by a mix of lysozyme and Triton and the fusion protein present captured and purified using Microspin GST columns (Amersham Biotech). Eluted fusion proteins at 10 μg/ml in 0.2 M NaHCO3 were used to coat the plate. Each serum was titrated on each construct and the endpoint titre determined. The relative binding of each serum to each fragment at this titre of serum is shown. Peak height s does not therefore represent their overall relative titre. [file 1742-4690-4-33-S3.tiff]

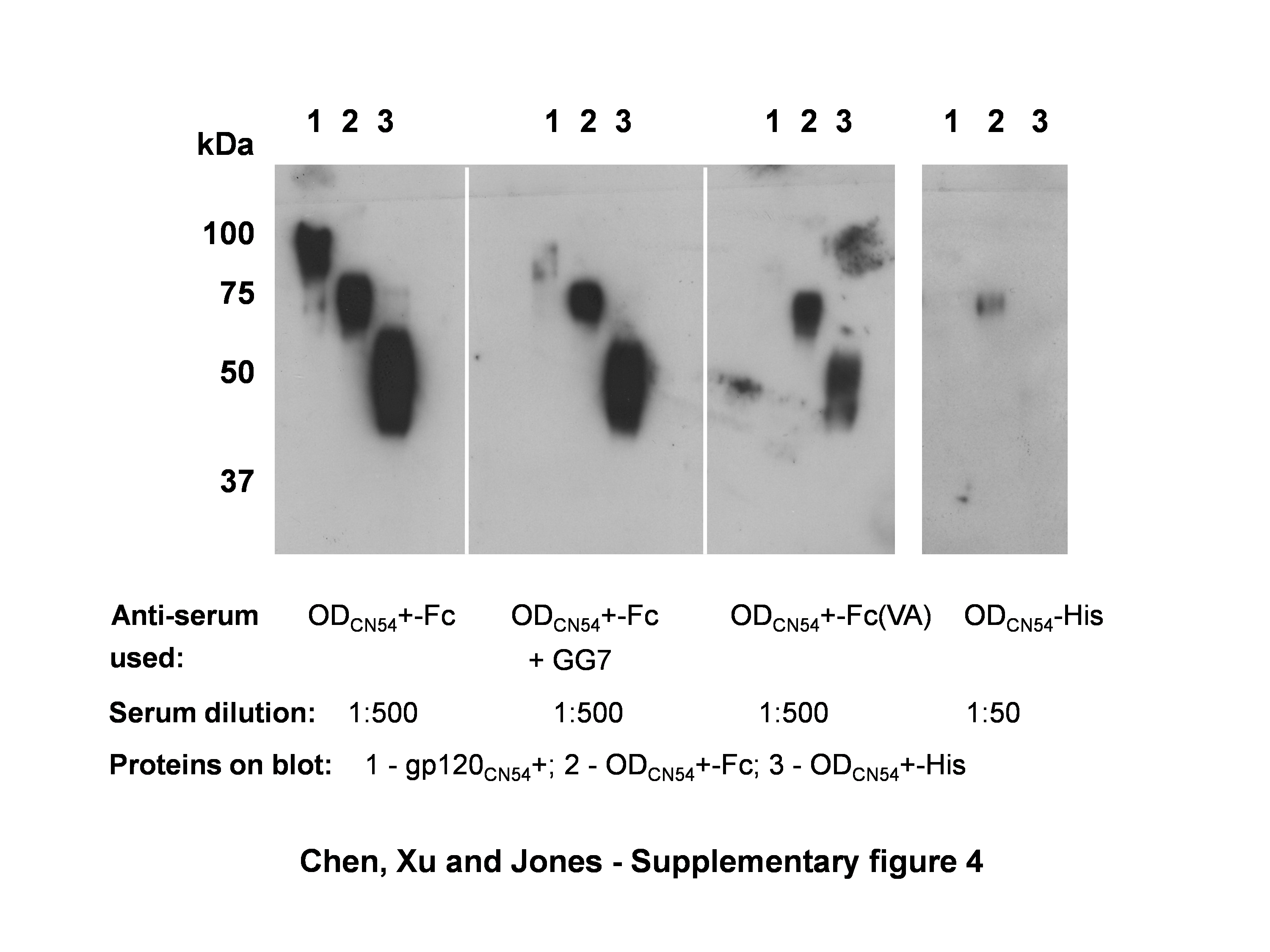

Supplement: Additional File 4 — Western blot of various sources of CN54 gp120 by the mouse sera raised by immunisation with only the outer domain, fused or not to Fc. The sera were used at the single dilution shown where blot intensity broadly paralleled the titre obtained by ELISA (Figure 6). Reaction between the serum raised by immunisation with ODCN54+-His and the targets was very poor and required a tenfold less dilution than the others. Reaction with ODCN54+Fc appears stronger as the band is much tighter (cf. Figure 5) although reaction with the smear of the cognate antigen is just visible (rightmost panel track 3). [file 1742-4690-4-33-S4.tiff]
